# Supplementary material for: Rapid turnover of life-cycle-related genes in the brown algae
Source: Genome Biol. 2019 Feb 14;20:35. doi: 10.1186/s13059-019-1630-6 (PMC6374913; doi:10.1186/s13059-019-1630-6)
Supplement: Supplementary file 3 — Protein alignments, corresponding Gblocks html file and CDS sequences for the lineage conserved gametophyte-biased genes. (ZIP 2109 kb) [file 13059_2019_1630_MOESM3_ESM.zip › Con_GA_alignments/Ec-12_007410.aa_ali.fasta-gb.htm]

Ec-12\_007410.aa.fasta.trans.aa\_ali.fasta


## Gblocks 0.91b Results

Processed file: **Ec-12\_007410.aa.fasta.trans.aa\_ali.fasta**  
Number of sequences: **2**  
Alignment assumed to be: **Protein**  
New number of positions: **386** (selected positions are underlined in blue)

```
                         10        20        30        40        50        60
                 =========+=========+=========+=========+=========+=========+
Ec-12_007410     MDDITRGPFSYEHRETAPPWSVGWLPTSPTMAEQHTDDAFSRPRE-LLLGDHAHTDQQKK
MSTRG.42973.1    MEDITRGPFSHEQRDTAPPWSVGWLVTPSATVEHPTDGVVSTPRGGMAHGEQDHLDPQKK
                 ############################################     ###########


                         70        80        90       100       110       120
                 =========+=========+=========+=========+=========+=========+
Ec-12_007410     KLFVGMAPKSAYEEDYRAVFEPFGTPIDIYVIRDRNGFSKGCAFVRYESVKSASDAIEAL
MSTRG.42973.1    KLFVGMAPKSAYEEDYRAVFAPFGKLVDIYVIRDRNGFSKGCAFVRYESAQSANDAIDAL
                 ############################################################


                        130       140       150       160       170       180
                 =========+=========+=========+=========+=========+=========+
Ec-12_007410     HDKHIMPGGFRTLVVTVADDRRGPQTSAVGTTTTRRDAVGSGDGLFRALSRPSSFMGSDS
MSTRG.42973.1    HDKHIMPGGFRTLVVTIADDRRSPHAAAASTATCHRDTLCSEDGLSRSLSRPASFLGGDG
                 ############################################################


                        190       200       210       220       230       240
                 =========+=========+=========+=========+=========+=========+
Ec-12_007410     GGGGTIPSLGNVVGQNRSTVTAPQAGATAFAPTVAPGSGPSSGLLSGMPYFLCGGNPPQG
MSTRG.42973.1    GGGGTISSTGSIVGPHRSSAPAPQTGAVAFGSTVAQGSTP-AGVLPGMPYFLCGGNPPQG
                 ########################################  ##################


                        250       260       270       280       290       300
                 =========+=========+=========+=========+=========+=========+
Ec-12_007410     TYVYYPYVPSSSVQHDTGSSAAPAGIGAKPHHAFSVGSPAVGNNDRCELGARGAQEDGYT
MSTRG.42973.1    TYVYYPYVPPSCSQPDAAVSVAPPEVSTKSHHAPSIGSHGVAN--VRGRGARGVQENGYV
                 ###########################################      ###########


                        310       320       330       340       350       360
                 =========+=========+=========+=========+=========+=========+
Ec-12_007410     GDRTVRKFGTGGPEERGRRWAKQSVGPTGANLFVYYLPGSLTDADLATAFAPFGEVLSAK
MSTRG.42973.1    DDGTVRRFSSGGPEEKGRRWAKQSVGPTGANLFVYYLPGSLTDADLATAFSPFGEVLSAK
                 ############################################################


                        370       380       390       400       410       420
                 =========+=========+=========+=========+=========+=========+
Ec-12_007410     VYYDRDTGESKGFGFVSYSKPDEAEAAISSMNGFFIGQKF--------------------
MSTRG.42973.1    VYYDRDTGESKGFGFVSYSTPEEAEAAISGMNGFFIGQKRLKVVRKRGQQADRYTDGGTR
                 #######################################                     


                        430       440       450       460       470       480
                 =========+=========+=========+=========+=========+=========+
Ec-12_007410     ------------------------------------------------------------
MSTRG.42973.1    RNGRGGDFGYAGSNSASGMAQAESGDVGHGGIHPPDDDNVTSEISEHRTLNGNGFQENLD
                                                                             


                        490
                 =========+======
Ec-12_007410     ---------------L
MSTRG.42973.1    FFSPEGLLGQSMARLL
```

```
Parameters used
Minimum Number Of Sequences For A Conserved Position: 2
Minimum Number Of Sequences For A Flanking Position: 2
Maximum Number Of Contiguous Nonconserved Positions: 8
Minimum Length Of A Block: 10
Allowed Gap Positions: None
Use Similarity Matrices: Yes
```

```
Flank positions of the 4 selected block(s)
Flanks: [1  44]  [50  220]  [223  283]  [290  399]  

New number of positions in Ec-12_007410.aa.fasta.trans.aa_ali.fasta-gb:  386  (77% of the original 496 positions)
```
